# Measurement of the Direct Impact of Hematophagous Flies on Feeder Cattle: An Unexpectedly High Potential Economic Impact

**DOI:** 10.3390/insects15100735

**Published:** 2024-09-24

**Authors:** Phoompong Boonsaen, Adèle Nevot, Sathaporn Onju, Clément Fossaert, Piangjai Chalermwong, Kornkanok Thaisungnoen, Antoine Lucas, Sophie Thévenon, Roungthip Masmeatathip, Sathaporn Jittapalapong, Marc Desquesnes

**Affiliations:** 1 Department of Animal Science, Faculty of Agriculture at Kamphaeng Saen, Kasetsart University at Kamphaeng Saen Campus, Nakhon Pathom 73140, Thailand; pboonsaen@gmail.com; 2 Centre de Coopération Internationale en Recherche Agronomique pour le Développement (CIRAD), UMR INTERTRYP, Bangkok 10900, Thailand; adele.nevot@gmail.com (A.N.); clement.fossaert@gmail.com (C.F.); antoine.lucas@agrocampus-ouest.fr (A.L.); marc.desquesnes@cirad.fr (M.D.); 3 Faculty of Veterinary Technology, Kasetsart University, Bangkok 10900, Thailand; 4 Department of Entomology, Faculty of Agriculture at Kamphaeng Saen, Kasetsart University, Nakhon Pathom 73140, Thailand; drohnaja18@gmail.com (S.O.); fagrrtm@ku.ac.th (R.M.); 5 Faculty of Veterinary Medicine, Kasetsart University, Bangkok 10900, Thailand; joy_piangjai@hotmail.com (P.C.); kornkanok.tha@gmail.com (K.T.); 6 INTERTRYP, Univ Montpellier, Centre de Coopération Internationale en Recherche Agronomique pour le Développement (CIRAD), Institut de Recherche pour le Développement (IRD), 34980 Montpellier, France; sophie.thevenon@cirad.fr; 7 Centre de Coopération Internationale en Recherche Agronomique pour le Développement (CIRAD), Unité Mixte de Recherche INTERTRYP (UMR INTERTRYP), 34398 Montpellier, France; 8 Center for Advanced Studies for Agriculture and Food, Kasetsart University Institute for Advanced Studies, Kasetsart University, Bangkok 10900, Thailand

**Keywords:** biting flies, mosquitoes, annoyance, production loss, weight gain, feed

## Abstract

**Simple Summary:**

Biting hematophagous dipterans are responsible for painful bites and blood spoliation; they induce behavioral modifications, anemia, and significant production losses in cattle. A feedlot of 100 feeder cattle would register a total loss of USD 16,000 within 5 months, which appears to be an unexpectedly huge loss caused by dipterans. Investing part of this money in fly control would probably be beneficial.

**Abstract:**

In addition to blood pathogen transmission, insects of the order Diptera affect livestock through visual and contact harassment; blood-feeders are responsible for painful bites and blood despoliation, generating behavioral modifications, anemia, and production losses. Knowledge of their economic impact is a basis for cost-effective control. Here, we measured the global impact of diptera insects by comparing two batches of six feeder cattle, one in the open air and the other protected by a mosquito net. The analytical data were insect density in the open air and, for feeder cattle, tail flick counts, hematocrit values (Ht), feed intake, feed conversion ratio (FCR), and live body weight gain (LBWG). Over a period of five months, the results showed significant losses in the LBWG of cattle exposed to insects, estimated at 8.0 ± 1.5 kg/month [2.7; 13.3], with a total loss reaching 40.0 ± 5.5 kg/head. Main diurnal insects were *Stomoxys* spp. and *Musca crassirostris*. There was a strong correlation between fly density and diurnal tail flicks. Night trapping and tail flicks showed a potentially important role of mosquitoes to be further explored. The Ht levels of exposed animals were 3–4% lower than those of controls. FCRs indicated that exposed animals needed 33% more dry matter intake/kg of LBWG. An economic assessment showed that dipterans were responsible for a 10–11% loss in LBWG during the main growing period of feeder cattle (10–15 months). A feedlot of 100 calves would register a total loss of USD 16,000 within 5 months, which appears to be an unexpectedly huge loss caused by dipterans. Investing part of this money into fly control would probably be beneficial.

## 1. Introduction

Insects belonging to the order Diptera are among the most important pests for livestock and humans [1]; the most common are the sucking flies of the genus *Musca*, and the most annoying for livestock are the blood-feeding dipterans, which are proliferating under massive monoculture production [2]. Hematophagous dipteran insects belong to the suborders Nematocera and Brachycera. Small-sized dipterans, such as mosquitoes (Culicidae), Culicoidea, Ceratopogonidae, and Simuliidae belong to the order Nematocera [1]. Larger hematophagous biting flies belong to the order Brachycera, such as Stomoxyine flies (tribe Stomoxyini), tabanids, tsetse flies, and hippoboscids, including sucking flies for which hematophagia is either obligatory (such as *Musca ventrosa*, *M. inferior*, and *M. crassirostris* [3,4]) or facultative (such as *Musca domestica*, *M. automnalis*, etc.). Among these insects, the most prevalent in livestock are sucking flies of the genus *Musca*, Stomoxyine flies, especially those belonging to the genus *Stomoxys* (stable flies), tabanids (especially those belonging to the genera *Tabanus*, *Chrysops*, and *Haematopota*), and mosquitoes. Apart from the physical act of taking a blood meal, these flies are also mechanical vectors of pathogens, including parasites, bacteria, and viruses [5,6].

Physical or chemical protections are generally ineffective and thus not consistent over time [7]. Unless bred indoors and/or under permanent mosquito net protection, livestock is permanently subjected to the annoyance, bite, and blood spoliation inflicted by diurnal and nocturnal hematophagous insects. Their impact on livestock has never been fully determined; however, based on the partial data available and estimations made for some types of insects, such as *Stomoxys* spp. and tabanids, it is generally accepted that biting flies are responsible for a huge loss in livestock production. In a very early study conducted in 1947 in Illinois, US, it was estimated that controlling tabanids through insecticide spray (every 3–4 days) might cause an additional weight gain of 9–13.5 kg per 5.5 weeks in free-pasturing cattle [8,9]. In a study conducted in the USSR from 1982–1984, it was estimated that tabanids were responsible for a decrease of 13% in dairy production; at that time, only insecticide spray was proposed to decrease their impact [10]. In French Guyana, in 1992, the daily weight gain of feeder cattle registered during the tabanids’ infestation season decreased from an average of 1200 g to 200 g [11]. Stomoxyine flies, such as *Hematobia* (horn flies) and *Stomoxys* (stable flies), are sometimes considered responsible for a loss of 50% in milk production [12] and for an important loss in weight gain in feeder cattle [13]. Other studies in the US have emphasized the predominant impact of horn flies in cattle, including beef and dairy cattle; an increase of 12.5 kg was obtained in calf weight on weaning in insecticide-protected animals versus the control group [14]. In another study carried out in the US, the losses due to stable flies were estimated per animal and year at 139 kg of milk for dairy cows and 6, 26, and 9 kg of live body weight for pre-weaning calves, pastured stockers, and feeder cattle, respectively [15]. While ignoring or underestimating the global impact of hematophagous flies, and since the available control methods are expensive and poorly efficient, fly control is a part of livestock management that is most often neglected. Entomological and socio-economic studies are required to stress the importance of these insects, and new methods are needed to complete their control.

Whichever specific control method program is adopted, integrated management is a basic tool to be applied first [7,16], such as general cleaning, the elimination of water (mosquito larval habitat) [17], and manure and vegetation waste elimination (Muscidae larval habitat) to avoid offering culture media for insect larvae [16]. However, killing adult insects remains necessary when facing a high seasonal peak of infestation, and new methods are currently under development to adapt insecticide-impregnated screens to attract and kill hematophagous flies [18]. Such tools might easily be adopted once farmers appreciate their potential benefits. A step before investing in a pest-control program is to evaluate the amount of money lost due to the pest.

In the present study, we compared the defense movements (tail flicks), hematocrit values, feed intake, growth performances, and feed efficiency in two groups of feeder cattle, which were kept free inside stables. One group was protected by a mosquito net and the other was kept in open-air conditions.

## 2. Material and Methods

### 2.1. Study Area and Equipment

This study was carried out at Kasetsart University, Kamphaeng Saen Campus, Nakhon Pathom Province, Thailand, between May 2016 and February 2017.

**Figure 1:**
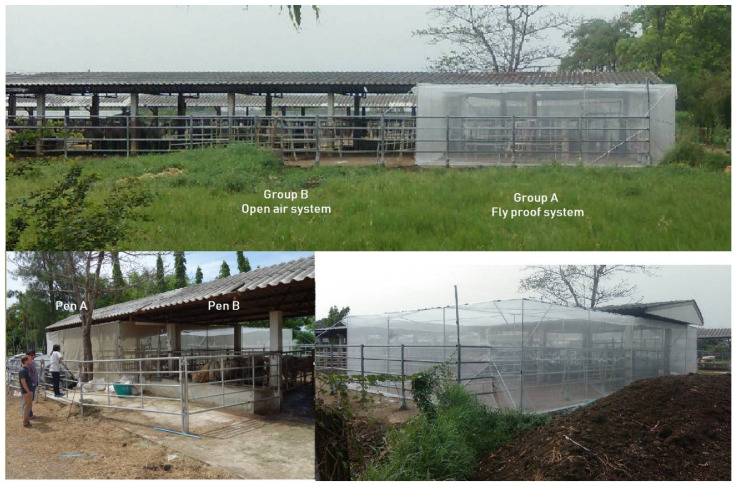
Pictures of the experimental devices; Group A and Pen A: fly-proof system; Group B and Pen B: open-air system.

Two stables (A and B) measuring 7 × 12 m (84 sqm each), with roofs covering a third of each, were delimited and separated 2 m apart. Each of these stables was equipped with a feed trough and a water trough. In the middle of each stable, 1 m under the roof, a device was installed to record temperature and hygrometry. Stable B was a normal open-air area. Stable A was entirely covered with a mosquito net (fly-proof system), which was installed one meter away from the fence and one meter away from Stable B (to avoid it being torn off by the animals), including a mosquito roof 2.5–3 m high and an additional entrance (airlock) to avoid introducing insects when coming in and out of the stable (Figure 1). The inside of the airlock was equipped with a fan oriented toward the entrance to avoid introducing flies when coming in and out while bringing feed or removing cowpat. This fan was turned on when using the entrance airlock.

Two fans were fixed in a high position under the roof inside each stable to blow the air toward the ground to lower the stable temperature, if necessary, to keep the temperature as close as possible in both stables. As a rule, it was established that fans would be turned on in a stable when its temperature was 2 degrees Celsius higher than the temperature of the other stable. The temperatures of both stables were recorded 3 times a day, in the morning (8:00 a.m.), at noon (12:00), and in the evening (5:00 p.m.). If flies were found inside the mosquito net stable, they were caught and destroyed each day during morning and evening checks. In both stables, cowpats were removed daily, in the morning and the afternoon, to avoid any fly larval development.

### 2.2. Animals Housing, Management, and Grouping

Two experiments were carried out successively, applying the same protocol, as follows: Experiment 1 from 10 May to 11 July 2016, and Experiment 2 from 7 September 2016 to 22 February 2017. For each experiment, 12 animals were selected out of a group of 30 weaned Kamphaeng Saen beef (KPS) bull calves [19] 6.5–12 months of age that was issued from the Animal Sciences Breeding Unit (Faculty of Agriculture at Kamphaeng Saen, Kamphaeng Saen Campus, Kasetsart University). At first, all calves were kept free in an open-air 300 sqm park and observed daily for 3 days by two technicians to score the attractivity and behavior of the animals vis-à-vis the flies. Attractivity was estimated using the mean of 3 repeated counts of the total flies visible on one side of each animal, morning and evening, for 3 days; animals exhibiting extreme values were rejected. Reactivity/passivity was estimated through the observation of defense movements; increasing intensity was scored from 0 to 4 at the same time as the insect counts in the presence of moderate fly density (5–10 flies visible on the animal), and the scores were defined as follows: 0: no reaction; 1: skin shaking only; 2: skin and head movements; 3: skin, head and tail movements; and 4: skin, head, tail, leg, and head movements. Animals exhibiting extreme reactivity (mean score > 3) or extreme passivity (mean score ≤ 1) were excluded from the experiment. Moreover, animals showing aggressive behavior toward each other or the manipulators were also rejected. Non-rejected animals were weighed before the selection was completed (using TAN Scale Model LP7110C-1T, VT Thepamnuay Scale Company Limited, Nakhon Pathom, Thailand). Age and weight were also considered, and animals with extreme values were excluded. The remaining candidate animals were submitted to parasitological, serological, and molecular blood tests for hemoparasites (see below). Only negative animals were kept for the experiment. Twelve bull calves were selected as described above and randomly split into two groups of 6 animals (Group A and B), considering their ages and weights, to constitute two groups exhibiting mean weights and ages as similar as possible. These animals were treated against intestinal and blood parasites with Aben-15^®^ (150 mg Albendazole; F.E. Pharma Company Limited^®^, F.E.Pharma Company Limited, Bangkok Thailand) and Ivomec^®^ (Merial Limited, Duluth, GA, USA) Plus (1% Ivermectin and 10% Clorsulon, Merial^®^ Inc., Duluth, GA, USA). Animals were introduced into a pen (A or B) at the end of week 2 of each experiment (the first two weeks being during the preliminary period when the 12 calves were kept together in open air). The animal study protocol was approved by The Animal Usage and Ethics Committee of Kasetsart University, Thailand (ACKU60-AGK-006 and date of approval on 21 December 2016).

### 2.3. Follow-Up of the Animals’ Health

From 30 days prior to the introduction of the cattle in the stables and every 2 weeks during all the experiments, blood samples were collected to check the following markers:

(i) the hematocrit value (pack cell volume, PCV%) using the capillary centrifugation method;

(ii) the presence of moving blood parasites (*Trypanosoma*, *filaria*) through microscopic observation of the buffy coat using the capillary centrifugation method [20];

(iii) the presence of blood parasites (*Anaplasma, Babesia, Trypanosoma, Theileria*) through microscopic observation of Giemsa-stained thin blood smears;

(iv) the presence of antibodies directed against *T. evansi* using the card agglutination test for Trypanosomes/*T. evansi* (CATT/*T. evansi*) and enzyme-linked immunosorbent assay against *Trypanosoma evansi* (ELISA *T. evansi*) [21,22];

(v) the presence of *Trypanozoon* DNA through PCR (Chelex^®^ prepared buffy coats) [23] using TBR primers with previously published protocols [24,25].

### 2.4. Feed and Feed Intake

The concentrate diet and fresh para grass (*Brachiaria mutica*) were fed *ad libitum* twice daily (at 07:00 a.m. and 05:00 p.m.). The ingredients and chemical compositions of the diets are shown in Table 1.

The weight of the hay and concentrate delivered were recorded daily in the morning and the afternoon, as well as the weight of the hay and concentrate remaining, which were recorded in the morning of the next day; additionally, to evaluate a potential effect on the feed ingestion rhythm of the animals, the food remaining before the afternoon delivery was visually scored from 0 to 3 and recorded for statistical analysis. At the beginning of the experiment, the first amounts of feed delivered were estimated based on the age and weight of the animals, but later on, this amount was increased based on the amount ingested the day before, plus 2–3 kg, so that a larger amount was present at all times and the feed was considered to be delivered ad libitum. Animals had permanent free access to feed and clean drinking water.

### 2.5. Insect Rapping and Identification

**Figure 2:**
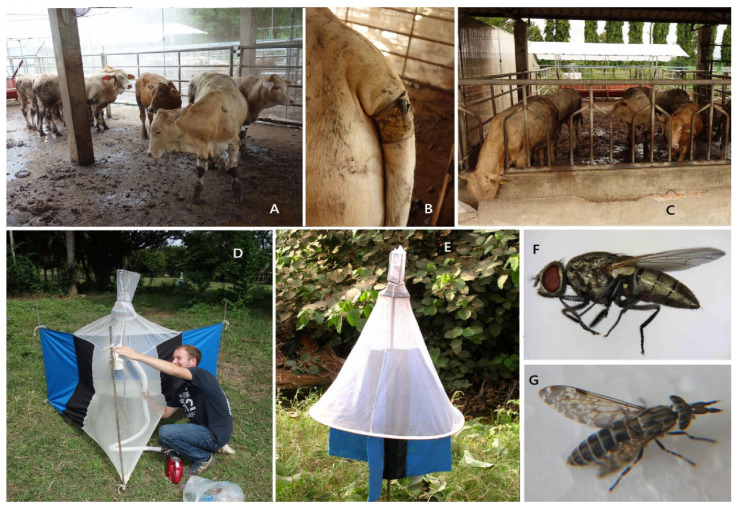
Pictures of feeder cattle under a mosquito net (**A**) with tail pedometer disposal (**B**) and in open air (**C**), Nzi trap collection (**D**), Vavoua trap (**E**), and some hematophagous flies: *Musca crassirostris* (**F**) and a tabanid: *Haematopota* sp. (**G**).

As mentioned in the introduction, the most important dipteran pests for livestock are large-sized hematophagous Brachycera flies; Vavoua and Nzi traps, which are fairly well-adapted to such flies, were used [26,27,28]. Once a week, in the pasture next to the stables (see the grass area in front of the stables in the upper picture, Figure 1), two Vavoua traps (Figure 2E) and one Nzi trap (Figure 2D) were set up on a line 60 m apart from 06:00 a.m. to 6:00 p.m., and the trapping-cages were installed, starting from 6:00 a.m. for 6 trapping-periods of 2 h; cages were then collected at 8:00, 10:00, 12:00, 14:00, 16:00, and 18:00 to evaluate the temporal distribution of flies throughout the day. Insects were identified using keys for *Musca*, tabanids, and Stomoxyine flies [29,30,31]. The total number of insects trapped in a day was used to evaluate the insect density in the study area. To demonstrate the annoyance effect of flies on cattle, the number of insects trapped during each of the 6 trapping periods was compared with the tail counts recorded during the same periods using a so-called “tail-pedometer” (a pedometer attached 20 cm from the basis of the tail; Figure 2B). Data analyses were conducted for “hematophagous flies” (*Stomoxys* spp. + *Musca crassirostris* + tabanids) and “common flies” (*Musca* spp. with the exception of *M. crassirostris*) (Figure 2F).

Once, in the midterm of Experiment 2, to evaluate the potential role of mosquitoes in the annoyance of cattle, a mosquito trapping session using a CDC trap [32] hung 2.5 m high in the center of Stable B was organized at night, from 6:00 p.m. on 21 November 2016 to 6:00 a.m. on 22 November 2016; all insects trapped were identified and counted [33,34]. In these experiments, insect trapping and identification were recorded to help identify the source of the annoyance of the cattle, and a complete study of fly seasonal variations and fly density linked with pedometer records has been detailed and published elsewhere [35].

### 2.6. Defense Movement Evaluation in Cattle

Defense movements of cattle to fend off insects include ear, tail, head, leg, and skin movements [36]; among these, tail flicks proved to be a significant marker of biting fly annoyance [37,38,39], reaching or passing 20 flicks per minute in the case of medium to high fly density in cattle or buffaloes [2].

To count tail defense movements, Fitbit^®^ pedometers were attached to the tails of the feeder cattle to collect “Pedometer Records” (PR). Fitbit zip^®^ pedometers (manufactured by FitBit Inc., San Francisco, CA, USA) are developed for sports and activity monitoring; they are waterproof and powered by disposable batteries, and they continuously record movements (“steps”). Step counts can be downloaded after Bluetooth synchronization using an electronic device (smartphone or computer) from the FitBit website. One pedometer was attached to the tail of each animal at a distance of 15–20 cm from the base of the tail, first using a regular gauze band covered by an elastic gauze band and later on, fixed with a sticky gauze band (Neotape 2^®^, Neo G Limited, Killinghall, UK). The pedometer records (PR) (so-called “steps” on the FitBit website) of animals in both groups were downloaded and saved three times a week using the Fitbit smartphone application for further data analysis. Continuous diurnal and nocturnal data obtained from the electronic devices were recorded from 10 May 2016 to 25 January 2017. Nocturnal PRs (from 6:00 p.m. to 6:00 a.m.) and diurnal PRs (from 6:00 a.m. to 6:00 p.m.) were downloaded and recorded separately to evaluate the relative impact of diurnal versus crepuscular and nocturnal insects. In this experiment, tail flicks were recorded to quantify the intensity of the annoyance during various periods of time, and thus to identify the source of the annoyance of the cattle, and a complete study and validation of this method with regard to insect trappings has been detailed and published elsewhere [35].

### 2.7. Statistical Analyses

Statistical analyses were carried out with program R version 3.6.0 [40] in an R-studio environment. The means are indicated with 95% confidence intervals. Comparisons of mean weight and age of the animals in Groups A and B at the beginning of Experiments 1 and 2 were carried out using *t*-tests, and the results were significant when the *p*-value was <0.05; the same method was used to compare feed intake, dry matter intake (DMI), and feed conversion ratios (FCRs).

Construction of the 95% confidence interval for the difference in mean body weight between Groups A and B (small-size groups, *n* < 30) was made using a t-table at the appropriate degree of freedom, according to a previously described method [41]. As recommended, the following assumptions were checked: (i) there is the homogeneity of the variances in the two groups; this was demonstrated by using Levene’s test, and it was accepted for *p*-values > 0.05; (ii) the data of the two groups are normally distributed, which was checked by using the Shapiro–Wilk test and was accepted for *p*-values > 0.05; and (iii) each value needs to be sampled independently from each other value (the case for individual animal weights in this study).

A comparison of the PCVs of Groups A and B was made using a two-way repeated measures ANOVA, and graphical representations were rendered using the package ggplot2 [42]. Two periods of time were defined in Experiment 1, and 3 periods were defined in Experiment 2 based on the graphical observation of the “low” or “high” difference of the PCVs between the 2 groups.

For weight comparisons, graphical representations were rendered using the package ggplot2 [42], and for analysis, following previously published recommendations [43], several models were tested, some using a maximum likelihood (ML) algorithm, but the one that seemed to best fit the data was the two-way repeated measures ANOVA [44]. This method was applied to 4 different periods based on the graphical observation of “high” or “low” insect densities recorded during Experiments 1 and 2, before and at the beginning of the screens setting (Period 1), and along 3 following periods until the end of the experiments (Periods 2–4).

In both cases (PCVs and weights), as recommended, the following assumptions were checked: (i) homogeneity of the variances in the two groups (demonstrated by using Levene’s test and accepted for *p*-values > 0.05); (ii) normality of the data (checked by using the Shapiro–Wilk test and accepted for *p*-values > 0.05); and (iii) individual data were collected independently of each other. The mean PCV and weight in Groups A and B were significantly different when the *p*-value was <0.05 [44].

## 3. Results

The fly-proof pen was built as shown in Figure 1, one meter apart from the open-air pen. The temperature and relative humidity records were always very close in both pens. The temperature inside the fly-proof stable was sometimes slightly lower than that of the open air, but never more than one degree Celsius or 5% relative humidity. Consequently, the inside fans were never used, and we concluded that the slight differences in temperature and relative humidity between the two pens did not interfere with this study.

### 3.1. Animals and Grouping

Half of the 30 animals were rejected as described in Materials and Methods. All animals tested proved to be negative for all hemoparasite detection tests and remained negative throughout the experiments. Twelve calves 6.8–12.2 months of age were selected in Experiment 1 and 8.7–10.8 months of age in Experiment 2. The mean age of the animals was 314 ± 34 days in Experiment 1 and 299 ± 15 days in Experiment 2; there was no significant difference between the two groups (A/B) in both experiments (all *p*-values of *t*-test > 0.80; Table 2).

Experiment 1 lasted 9 weeks, from 10 May to 11 July 2016; the animals were all together during the first two weeks, and the animals in Group A entered the fly-proof device at the end of week 2 and spent 7 weeks under fly-proof conditions (week 3–9).

Experiment 2 lasted 24 weeks, from 7 September 2016 to 22 February 2017; the animals were all together during the first two weeks, and the animals in Group A entered the fly-proof device at the end of week 2 and spent 22 weeks under fly-proof conditions (week 3–24) (Figure 2A,C).

### 3.2. Insect Trapping and Identification

**Figure 3:**
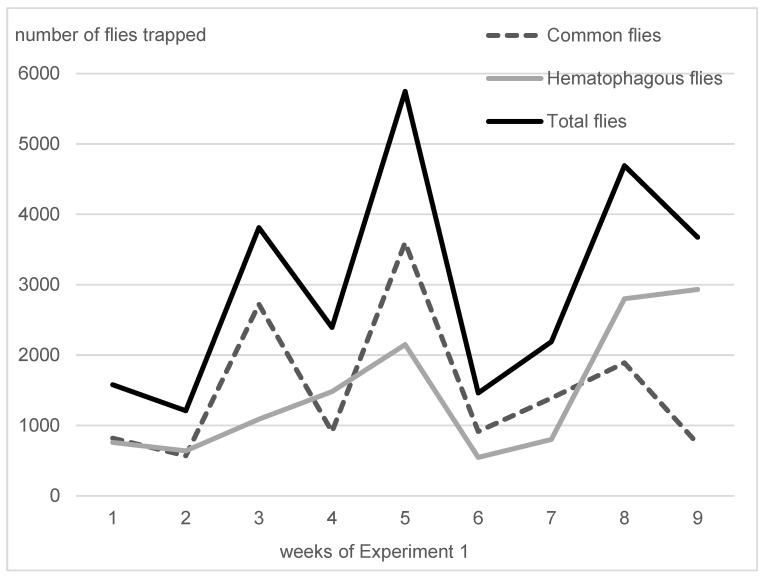
Fly densities observed during Experiment 1.

**Figure 4:**
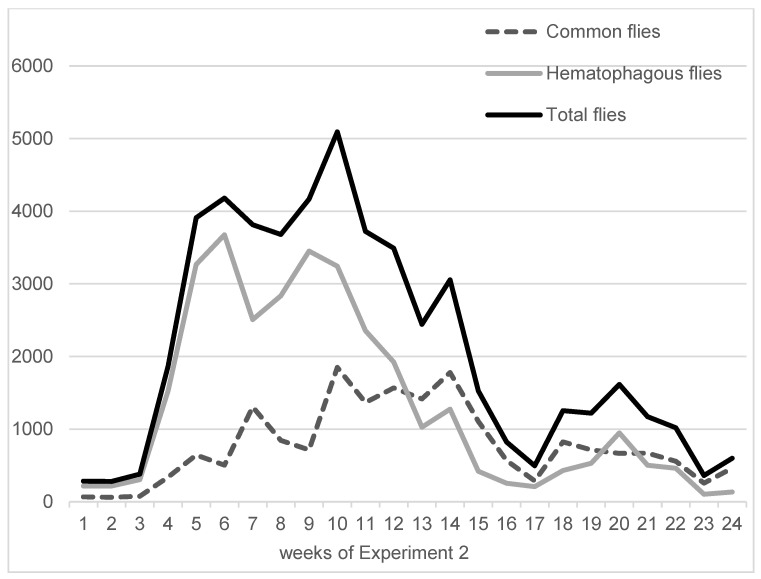
Fly densities observed during Experiment 2.

On average, in Experiment 1, hematophagous flies (*Stomoxys* spp. + *M. crassirostris* + tabanids) were in close proportions with sucking flies (*Musca* spp. excluding *M. crassirostris*, with 49.3% of the total catches), but they represented two-thirds of the total flies in Experiment 2 (63.1%). On average, the trapped hematophagous flies were dominated by *Stomoxys* spp. and *M. crassirostris*, a non-biting but obligatory blood-sucking fly that is highly prevalent in this area [4], while tabanids remained in low numbers. Insect trapping results from Experiments 1 and 2 are presented in Figure 3 (Experiment 1, 9 weeks, from 10 May to 11 July 2016) and Figure 4 (Experiment 2, 24 weeks, from 7 September 2016 to 22 February 2017). The daily total number of insects trapped in the three traps was between 547 and 2933 in Experiment 1, while it was between 104 and 3676 in Experiment 2.

Night trapping using a CDC trap showed significant mosquito activity, with a total of 2741 mosquitoes caught during a single night from 21 to 22 November 2016. Ninety percent of the mosquitoes were trapped between 06:00 p.m. and 12:00 p.m., indicating significant activity in the late evening and early night. Apart from mosquitoes, no other significant number of pest insects was caught at night.

### 3.3. Comparison of Mean Hematocrit Values

Hematocrit values (quantified every two weeks using the pack cell volume (PCV)) were considered good indicators of blood despoliation caused by hematophagous flies. The PCVs of the two groups of animals evolved as indicated in Figure 3. The mean PCVs of Groups A and B established at the beginning of the experiments (week 1) were not significantly different in Experiments 1 (*p*-value 0.17) and 2 (*p*-value 0.94). The mean hematocrit values of Group B became significantly lower than that of Group A in both experiments.

**Figure 5:**
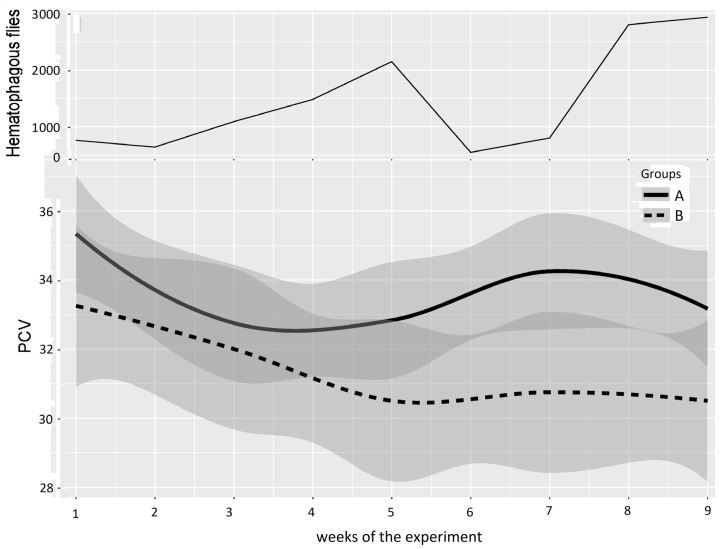
Mean PCV (95% CI) in Groups A and B and hematophagous fly densities in Experiment 1, from weeks 1 to 9. The broad lines indicate smooth mean values, the thin solid line indicates insect density, the bold solid line represents Group A, and the bold long dashed line represents Group B. Shaded parts correspond to the confidence interval of the means (at 95%).

In Experiment 1 (Figure 5 and Table 3), based on a “low” or “high” difference in the mean PCV in Groups A and B observed in Figure 5, periods were defined as Period 1: weeks 1–4, with no or low difference between Groups A and B, and Period 2: weeks 5–9, with a greater difference between Groups A and B. For Period 1, the results of the two-way repeated measures ANOVA between Groups A and B provided a *p*-value of 0.175, leading to a non-significant difference between Groups A and B at the beginning and early stages of Experiment 1. Conversely, the results of the two-way repeated measures ANOVA between Groups A and B provided a *p*-value of 0.001 for Period 2, which indicates a significant difference between Groups A and B.

**Figure 6:**
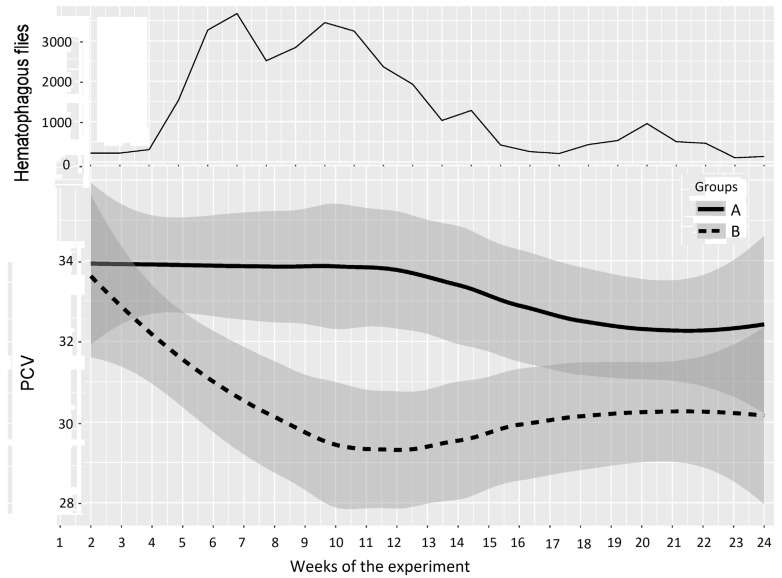
Mean PCV (95% CI) in Groups A and B and hematophagous fly densities in Experiment 2, from weeks 1 to 24. The broad lines indicate smooth mean values, the thin solid line indicates insect density, the bold solid line represents Group A, and the bold long dashed line represents Group B. Shaded parts correspond to the confidence interval of the means (at 95%).

In Experiment 2, PCV data are represented in Figure 6 and Table 4; the experiment was split into three periods of low, high, and medium differences between the two groups, as follows: Period 1: weeks 1–5 (before and just after Group A entered the fly-proof stable), Period 2: weeks 6–14 (high difference), and Period 3: weeks 15–24 (medium difference between the two groups). For the first period, the results of the two-way repeated measures ANOVA provided a *p*-value of 0.452, which showed a non-significant difference between Groups A and B. However, the results of the two-way repeated measures ANOVA between Groups A and B provided *p*-values of 0.000 and 0.006 for the second and third periods, which demonstrated highly significant differences between the PCVs of Groups A and B.

Overall, these results demonstrated that fly exposure significantly affected the PCV of the exposed animals, with mean PCV values 3–4% below the control group. In Experiment 2, the animals tended to recover their normal values when the fly density decreased. However, in the two experiments, the PCVs of the test groups never reached the levels of those of the control groups before the end of the experiments (the final PCV of Group B was 30.5% versus 33.2% in Group A in Experiment 1 and 30.2% versus 32.8% in Experiment 2).

### 3.4. Comparison of Mean Cattle Weight

**Figure 7:**
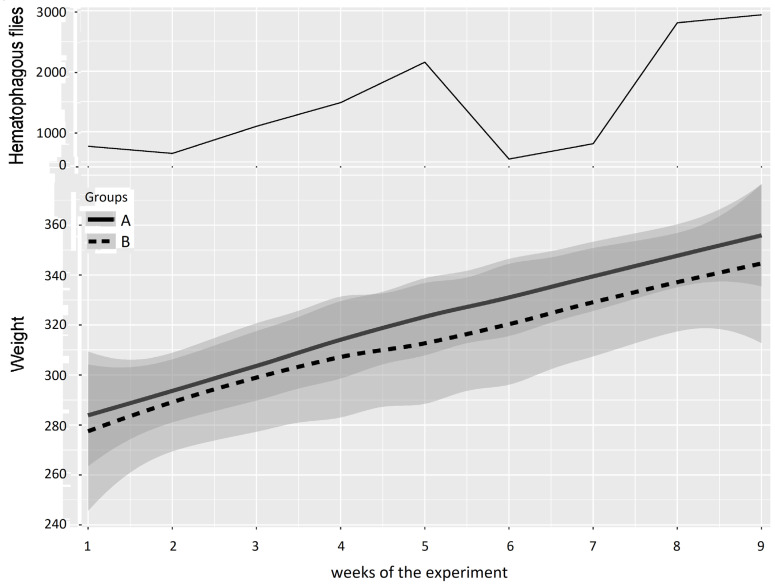
Mean cattle live body weight (95% CI) in Groups A and B and hematophagous fly densities in Experiment 1. The broad lines indicate smooth mean values, the thin solid line indicates insect density, the bold solid line represents Group A, and the bold long dashed line represents Group B. Shaded parts correspond to the confidence interval of the means (at 95%).

The mean weight of the cattle in Groups A and B and the number of hematophagous flies trapped per week during Experiment 1 (from 10 May 2016 to 11 July 2016) are reported in Figure 7. At the beginning of the experiment, the two groups showed similar mean weights (Group A: 284 ± 31 kg, Group B: 278 ± 45 kg), but when the insect density increased (weeks 3–5), the difference between the mean weights of Groups A and B increased to reach a maximum of 12.5 ± 4.9 kg in favor of Group A in week 5 at the peak of insect density (2149 hematophagous insects trapped in one day).

The results of the two-way repeated measures ANOVA on cattle weight in Experiment 1 are presented in Table 5. Weeks 1 and 2 were grouped for the first period (before Group A entered the fly-proof stable), weeks 3 to 5 were grouped for the second period (high fly density), weeks 6 and 7 were grouped for the third period (low fly density), and weeks 8 and 9 were grouped for the fourth period (high fly density). The results of the two-way repeated measures ANOVA between Groups A and B provided a *p*-value > 0.05 for the four periods; consequently, no significant difference could be demonstrated, although the mean weight of Group B was consistently and increasingly below that of Group A.

**Figure 8:**
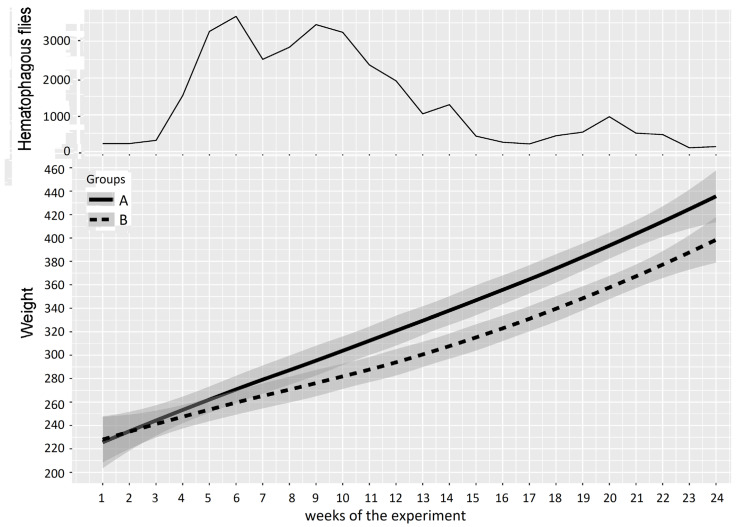
Mean cattle live body weight (95% CI) in Groups A and B and hematophagous fly densities in Experiment 2. The broad lines indicate smooth mean values, the thin solid line indicates insect density, the bold solid line represents Group A, and the bold long dashed line represents Group B. Shaded parts correspond to the confidence interval of the means (at 95%).

Experiment 2: the mean weight of cattle in Groups A and B and the number of hematophagous flies trapped per week during Experiment 2 (from 7 September 2016 to 22 February 2017) are reported in Figure 8. At the beginning of the experiment, the two groups showed similar mean weights (Group A: 223 ± 38 kg, Group B: 224 ± 26 kg), but when the insect density increased (weeks 3–24), the average growths in Groups A and B deviated and became significantly different. The mean loss of LBWG of Group B was around 1.8 kg/week (0.6–3.1) and was variable from one period to another, reaching 36.3 kg over 15 weeks (week 3–17) and up to 40.0 kg over 20 weeks (W3–24), which can be summarized as a mean loss of LBWG of 8.0 ± 1.5 kg/month (2.7;13.3). In week 24, the loss of LBWG reached 10.6% of the mean LBW of animals in Group B (the mean weight of animals in Group B was 378 kg at that time, versus 418 kg in Group A). The results of the two-way repeated measures ANOVA on cattle weight in Experiment 2 are summarized in Table 6.

Four periods were defined as follows: weeks 1–3 were grouped for the first period (prior to and just after Group A entered the fly-proof stable), weeks 4–13 were grouped for the second period (Group B was exposed to high fly density), weeks 14–20 were grouped for the third period (low fly density), and weeks 21–24 were grouped for the fourth period (relapse of high fly density). The two-way repeated measures ANOVA for cattle weight between Groups A and B provided a *p*-value > 0.05 (0.971) for the first period; consequently, no significant difference could be demonstrated between the mean weights of Groups A and B at the beginning of the experiment. Conversely, the results of the two-way repeated measures ANOVA between Groups A and B provided *p*-values < 0.05 for the three following periods, during which Group B alone was exposed to fly activity, demonstrating a significant difference between the mean weights of Groups A and B.

In the 22nd week of the experiment, after the second peak of hematophagous flies, the difference between the mean weights of Groups A and B was 40 ± 5.5 kg in favor of Group A.

### 3.5. Feed Conversion Ratio

The weights of the concentrate, hay, and dry matter intake (DMIs) in Experiment 1 and 2 are summarized in Table 7. Feed intake was not significantly different in Groups A and B in both experiments (all *p*-values > 0.45). The mean weekly feed conversion ratios (FCRs) were calculated as the rates of weekly dry matter intake (DMI) and weekly weight gain.

In Experiment 1, the mean FCR of Group B was greater than that of Group A, but not significantly. However, the mean FCRs were significantly different in Experiment 2 (*p* < 0.01). On average, in Group A (protected from flies), every kg of weight gain needed 4.97 ± 0.50 kg of DMI, while under fly exposure, in Group B, 6.62 ± 1.19 kg of DMI was needed. Consequently, the feed required to obtain 1 kg of weight gain was increased by 1.65 kg of DMI for animals exposed to flies, which represented a total need of 133% of the DMI needed in the control group.

With regard to the rhythm of feed ingestion, the remaining feed scores in the afternoon (indicating the amount of food not ingested during daytime) were slightly higher in Group B than Group A for concentrate (1.57 ± 0.16 > 1.53 ± 0.15), as well as for hay (1.38 ± 0.16 > 1.32 ± 0.16); however, these differences were not statistically significant in Experiment 1. In Experiment 2, the afternoon remaining feed scores of Group B were slightly lower than those of Group A for concentrate (1.59 ± 0.08 < 1.62 ± 0.08) but higher for hay (1.46 ± 0.09 > 1.40 ± 0.09); again, differences were not statistically significant, indicating that overall, the fly pressure on Group B was not high enough to divert the animals from feeding during the daytime.

### 3.6. Defense Movement Evaluation in Cattle (Experiment 2)

Overall, in Experiment 2, the daily tail pedometer records (TPRs) of Group A revealed a very low frequency of tail flicks or “steps”, always below 200 beats per 2 h during the daytime, with the maxima at the time of feed delivery and in the early morning and late afternoon and very low frequency recorded at night, generally below 50 beats per 2 h. Between September 2016 and February 2017, the mean daily TPRs recorded in Group A remained below 800 (Table 8), with a mean number of diurnal and nocturnal beats of 573 ± 69 and 164 ± 24, respectively. These records may be considered the basic activity of cattle in the absence of fly annoyance during this period and under these feeder cattle breeding conditions. The rate of nocturnal “steps” was low and stable, representing around 22% ± 1% of the total TPRs.

All data from Group B were highly significantly different from those of Group A (*p* < 0.05); thus, TPRs generally revealed a high frequency of tail flicks in the morning (frequently above 1000 steps/2 h) and up to 2500 steps/2 h or more in the evenings, with a strong correlation between the “steps” recorded and peaks of hematophagous fly density, which corresponds with the bimodal activity of the insects during this season [35]. Overall, in Group B, the mean daily diurnal TPRs reached 5333 ± 2044 beats (nine times higher than that of Group A), with a high and variable rate of nocturnal TPRs ranging from 15 to 74% and averaging 40% ± 15% (Table 6), which was 22 times higher than that of Group A. To evaluate the number of beats due to insects, we deducted the TPRs of Group A (beats due to the normal basic activity of cattle) from the TPRs of Group B (beats due to the basic activity of cattle + defense movements to the insects) (Table 6, last column), which led to mean additional numbers of beats due to insects of 3382 ± 1855 at night and 4761 ± 2106 in the daytime. These data suggest significant cattle annoyance at night (on average 40% of the total defense movements of cattle per 24 h). The insects trapped during one night of trapping using a CDC trap hung in the pen of Group B in November 2016 numbered 3120 specimens, which were principally composed of mosquitoes, with 2741 specimens (88%), while very few other dipteran pests were trapped, including 16 *Stomoxys* spp. (0.5%) and 24 common flies (0.8%). Other trapped insects were principally small coleopterans (6.8%) and lepidopterans (3.54%), as well as very low numbers of hymenopterans (0.4%) and hemipterans (0.1%). From these scores, it was strongly hypothesized that mosquitoes were responsible for the high numbers of tail pedometer beats recorded at night in Group B. A more detailed analysis of these data and results was published separately [35].

## 4. Discussion

Fly densities, peculiarly hematophagous flies, were quite variable during the two experiments; however, in both cases, they exhibited two serial peaks, which induced fluctuations in the recorded impact parameters. Overall, fly densities were considered to be high (>400 hematophagous flies/trap/day) during half of Experiment 1 (weeks 4–5 and 8–9) and Experiment 2 (weeks 4–14 and 18–22); they were lower in other periods. The recorded impact on cattle might then be limited to half of the experimental periods, thus, a higher impact may be expected in the case of higher or more constant fly density.

The direct impact of flies on cattle behavior was obvious from the tail pedometer records (TPRs), with, on average, more than 12 times more “steps” recorded in Group B (mean 24 h TPR 8879 ± 3545) versus Group A (736 ± 91) in Experiment 2; however, a part of these defense movements was due to nocturnal insects, as discussed below, when splitting diurnal and nocturnal TPRs.

In addition to the tail flicks, the feed intake could be affected by the flies’ annoyance; however, in the conditions of our experiments, the feed intake was not significantly different in the two groups, both in terms of feed ingestion rhythm and the total weight of the feed and dry matter ingested. However, the feed conversion ratio (DMI/weight gain), was higher in the exposed group, with a strong statistical significance in Experiment 2 (Group B needed 133% of the DMI needed by Group A for a similar weight gain), allowing us to conclude that decreased feed efficiency was induced by the insects’ annoyance. Similar results had been previously obtained in four-way mixed cattle feedlots (mean weight of 272 kg at the beginning of the experiment) under the pressure of the natural *Stomoxys* spp. population in Nebraska [45].

Due to blood cell ingestion, hematophagous flies are expected to impact the hematocrit of exposed cattle; indeed, a significant impact was recorded in Group B, especially in Experiment 2 (lasting longer than Experiment 1), in which the mean PCVs of the exposed animals reached a value 3–4% below that of the control group, which was synchronized with the high fly density periods (in Experiments 1 and 2) and showed an immediate direct effect of hematophagous flies on this cattle health blood parameter.

The live body weight gain (LBWG) of cattle was also impacted by high fly densities, as shown by the highly significant results obtained when comparing the LBWG of Groups A and B. In Experiment 2, the mean loss of LBWG of Group B was 8.0 ± 1.5 kg/month [2.7; 13.3]. This loss was recorded under a “medium” fly pressure (apparent density per trap < 500 in both experiments); greater losses can be expected under higher fly pressure.

A direct link between LBWG and fly density is not easy to draw, since other parameters impact weight gain, such as compensatory growth after a period of reduced growth [46], habituation of the cattle to high fly density after a period of exposure [47], climatic conditions [48], interactions among cattle in a group, etc. However, the global impact of flies on the weight gain measured in this study calls for serious attention in terms of loss of income. To use a universal unit, we calculated the shortfall of the weight gain on feeder cattle, taking the results of Experiment 2 as a model. In this experiment, within 20 weeks of exposure to flies (weeks 3–22), the loss of LBWG in Group B reached 40.0 ± 5.5 kg. This loss of LBWG amounted to 10.6% of the LBW of exposed cattle, which was lost within five months during their main growing period (10–15 months of age). When considering the current market value of KPS beef LBW in Thailand (estimated at around 125 THB/kg or 4 USD/kg in 2019), a feedlot of 100 calves would amount to a total loss of around 4 tons of LBW, for a value of USD 16000.

In a study carried out in the US, based on the cattle inventories and average prices for 2005–2009 and median monthly infestation levels, national losses were estimated to be USD 360 million for dairy cattle, USD 358 million for cow-calf herds, USD 1268 million for pastured cattle, and USD 226 million for cattle on feed, for a total impact on U.S. cattle industries of USD 2211 million per year. We would hardly extrapolate our results to a whole country, but, depending on farming conditions, the economic impact of flies might be very serious and could be roughly estimated to be around 10% of cattle LBW lost during five months of medium to high fly density. Our results are quite consistent with previous estimations made in the US (West Central Nebraska), in which, on average, losses of weight gain recorded in grazing yearling cattle exposed to *Stomoxys calcitrans* were estimated to be around 0.2 kg/day [49]; extrapolated to a period of five months, the loss would amount to a total of 30 kg of LBW/head. Because our results (a total loss of 40 kg LBW/head) were obtained over only 5 months, it is possible that the longer observation made by Campbell et al. [49] somehow “buffered” the effects of flies over time, or the additional effect of the mosquitoes was registered in our case. In another study, in free pasturing cattle, the mean additional weight gain in insecticide-treated animals was 8.2 kg per month, which represents 41 kg for 5 months [8,9]; the results, again, are very close to ours.

The present study was designed to measure the impact of hematophagous flies on cattle. However, due to the fly-proof system setup, our device automatically included protection against mosquitoes, and the impact measured on cattle kept in the open air also included the effects of flies and mosquitoes on cattle. Evaluating the potential relative role of mosquitoes on cattle, based on the total numbers and rates of diurnal versus nocturnal tail pedometer records in Experiment 2, cattle behavior was seriously impacted by insects at night, which is evidenced by an average of 3545 beats/night, representing 40% of the additional beats recorded per 24 h in exposed animals versus the controls (Table 6). Whether there is a direct link between the number of tail flicks (or TPRs) and the loss of LBWG is a difficult point to conclude. Responsibility for the losses measured in this study may be shared between hematophagous flies during the daytime and mosquitoes at night, but the relative impact of flies and mosquitoes would be difficult to establish unless a specific experimental study is designed for that purpose. Most of the authors considered mosquitoes to be negligible pests for livestock, as said by Hill [12]: “The feeding adults will take some blood and cause some irritation, but not usually a great deal”. The annoyance and abundance of mosquitoes are certainly underestimated because they occur at night, after the end of working hours. The potential impact of mosquitoes on livestock should be reconsidered in light of our results. As an example, in dairy cattle farms in Thailand, where farmers’ habitations are very close to cattle pens and stables, the animals’ owners very often complain about significant mosquito annoyance for themselves and their cattle. This is a topic on which limited investigations have been carried out so far. In that aspect, the use of tail pedometers proved to be very useful by providing continuous nocturnal and diurnal data that can easily be split to analyze relative diurnal and nocturnal pest attacks.

## 5. Conclusions

These experiments allowed us to demonstrate the highly significant impact of flying insects on cattle, including (i) a high loss of energy evidenced by tail flick pedometer records, showing, during the daytime, 12 times more tail flicks in cattle exposed to flies, (ii) red cell depletion evidenced by a significantly decreased PCV in exposed animals of 3–4% below the control group, (iii) decreased cost–benefit on the feed due to a significantly increased (by 33%) feed conversion ratio (FCR) in exposed cattle, (iv) a significant loss (averaging 10–11%) of LBWG in KPS beef feeder cattle 10–15 months of age, accounting for a money loss of USD 160 within 5 months, and (v) a non-negligible potential impact of mosquitoes on cattle, as evidenced by an important increase in tail flick defense movements at night (averaging 22 times more TPRs than non-exposed animals), requiring further evaluation.

Overall, in the 5-month study in Experiment 2, diurnal TPRs represented 60% of the total TPRs in exposed animals, suggesting a predominant impact of hematophagous flies on cattle while leaving room for the potential role of mosquitoes in total losses. The total loss of LBWG registered in this study was equivalent to 10–11% of the cattle LBW, which was lost within a season of insect activity; a part of this money could be fruitfully invested in fly control, providing that control methods are safe and cost-efficient.

## Figures and Tables

**Table 1:** Feed ingredients (as is basis) and chemical compositions (dry matter basis) of concentrate diet and para grass (provided by the purchaser).

Items	Concentrate	Para Grass
Feed ingredients (%)		
Molasses	8.80	
Urea	1.50	
Cassava chip	29.40	
Solvent-extracted palm kernel meal	29.40	
Expeller-pressed palm kernel meal	19.60	
Dehull soybean meal	4.90	
Ground corn	4.90	
Premix (Beef) ^1/^	0.50	
Sulfur	0.05	
Salt	0.50	
Dicalcium phosphate (Nuraphos-18)	0.50	
Chemical compositions (%)		
Dry matter, DM	89.30	23.10
Crude protein, CP	16.50	6.70
Ether extract, EE	4.20	–
Ash	5.50	70.80
Neutral detergent fiber, NDF	–	38.70
Acid detergent fiber, ADF	–	5.40
Acid detergent lignin, ADL	–	11.10
Calcium, Ca	1.20	–
Phosphorus, P	0.60	–

^1/^ Agromix beef No. 46: vitamin A = 2,160,000 IU, vitamin B3 = 100,000 IU, vitamin E = 5000 IU, Mn = 8.5 g, Zn = 6.4 g, Cu = 1.6 g, Mg = 16 g, Co = 320 mg, I = 800 mg, Se = 32 mg.

**Table 2:** Mean cattle age and weight of Groups A and B at the beginning of Experiments 1 and 2 and *p*-values of *t*-tests.

Experiments	Group A	Group B	*p*-Value (*t*-Test)
Experiment 1			
Mean body weight ± 95% CI (kg)	284 ± 31	278 ± 45	0.78
Mean age ± 95% CI (days)	311 ± 58	318 ± 59	0.84
Experiment 2			
Mean body weight ± 95% CI (kg)	223 ± 38	224 ± 26	0.93
Mean age ± 95% CI (days)	300 ± 24	299 ± 27	0.94

Mean weight of the animals was 281 ± 22 kg in Experiment 1 and 223 ± 19 kg in Experiment 2; there was no significant difference between the two groups (A/B) in both experiments (Table 2) (*p*-values of *t*-test > 0.75). Experiment 1 was stopped after 9 weeks because of violent interactions between bulls, probably due to a too-large age range; for Experiment 2, more age-similar calves were selected (8.7–10.8 months of age).

**Table 3:** Results of two-way repeated measures ANOVA on PCVs for Experiment 1.

Source of Variation	F Statistic	F Critical	*p*-Values
Time (Weeks 1–4 for the first period)			
Groups (A vs. B)	1.981	4.351	0.175
Time (Weeks 5–9 for the second period)			
Groups (A vs. B)	13.143	4.171	**0.001**

In bold: statistically significant result.

**Table 4:** Results of two-way repeated measures ANOVA on PCVs for Experiment 2.

Source of Variation	F Statistic	F Critical	*p*-Values
Time (Weeks 1–5 for the first period)			
Groups (A vs. B)	0.580	4.171	0.452
Time (Weeks 6–14 for the second period)			
Groups (A vs. B)	15.468	4.085	**0.000**
Time (Weeks 15–24 for the third period)			
Groups (A vs. B)	8.225	4.034	**0.006**

In bold: statistically significant results.

**Table 5:** Results of two-way repeated measures ANOVA on weight for Experiment 1.

Source of Variation	F Statistic	F Critical	*p*-Values
Time (Week 1–2 for the first period)			
Groups (A vs. B)	0.161	4.351	0.693
Time (Week 3–5 for the second period)			
Groups (A vs. B)	0.333	4.171	0.568
Time (Week 6–7 for the third period)			
Groups (A vs. B)	0.413	4.351	0.528
Time (Week 8–9 for the fourth period)			
Groups (A vs. B)	0.566	4.351	0.461

**Table 6:** Results of two-way repeated measures ANOVA on weight for Experiment 2.

Source of Variation	F Statistic	F Critical	*p*-Values
Time (Weeks 1–3 for the first period)			
Groups (A vs. B)	0.001	4.171	0.971
Time (Weeks 4–13 for the second period)			
Groups (A vs. B)	7.012	3.963	**0.009**
Time (Weeks 14–20 for the third period)			
Groups (A vs. B)	12.560	3.978	**0.001**
Time (Weeks 21–24 for the fourth period)			
Groups (A vs. B)	7.880	4.085	**0.008**

In bold: statistically significant results.

**Table 7:** Summary of statistical analysis of FCRs in Groups A and B in Experiments 1 and 2 using *t*-test.

Experiment 1 (9 weeks)	Group A	Group B	diff A-B	*p*-Values
Total concentrate ingested (kg)	1422	1420	2	0.97
Total grass ingested (kg)	1043	1045	−2	0.96
Total dry matter intake (DMI) kg	2860	2875	−15	0.95
Mean weekly FCR	5.65	6.36	−0.71	0.13
**Experiment 2 (24 weeks)**	**Group A**	**Group B**	**diff A-B**	***p*- ** **Values**
Total concentrate ingested (kg)	5294	5364	−70	0.49
Total grass ingested (kg)	4694	4704	−10	0.95
Total dry matter intake (DMI) kg	6453	6526	−73	0.89
Mean weekly FCR	4.97	6.62	**−1.65**	**0.01**

In bold: statistically significant results.

**Table 8:** Mean diurnal and nocturnal TPRs of Groups A and B in Experiment 2.

Tail Pedometer Records (TPR)	Group A	Group B	*p*-Value (*t*-Test)	Group B -Group A
Mean diurnal TPRs ± 95% CI	573 ± 69	5333 ± 2044	0.002	4761 ± 2106
Mean nocturnal TPRs ± 95% CI	163 ± 24	3545 ± 1856	0.008	3382 ± 1855
Mean 24-h TPRs ± 95% CI	736 ± 91	8879 ± 3545	0.003	8143 ± 3068
Percentage of diurnal TPRs	78% ± 1%	61% ± 14%	0.019	60% ± 15% *
Percentage of nocturnal TPRs	22% ± 1%	39% ± 14%	0.019	40% ± 15% *

* Established on the mean monthly results.

## Data Availability

Data are available upon request from the corresponding author.
